# Protective Mechanism of KIOM-4 in Streptozotocin-Induced Pancreatic *β*-Cells Damage Is Involved in the Inhibition of Endoplasmic Reticulum Stress

**DOI:** 10.1155/2011/231938

**Published:** 2010-09-16

**Authors:** Rui Zhang, Jin Sook Kim, Kyoung Ah Kang, Mei Jing Piao, Ki Cheon Kim, Jin Won Hyun

**Affiliations:** ^1^School of Medicine, Jeju National University, Jeju-si 690-756, Republic of Korea; ^2^Diabetic Complication Research Center, Division of Traditional Korean Medicine Integrated Research, Korea Institute of Oriental Medicine, Daejeon 305-811, Republic of Korea

## Abstract

Endoplasmic reticulum stress-mediated apoptosis plays an important role in the destruction of pancreatic *β*-cells and contributes to the development of type 1 diabetes. The present study examined the effect of KIOM-4, a mixture of four plant extracts, on streptozotocin- (STZ-) induced endoplasmic reticulum (ER) stress in rat pancreatic *β*-cells (RINm5F). KIOM-4 was found to inhibit STZ-induced apoptotic cell death, confirmed by formation of apoptotic bodies and DNA fragmentation. STZ was found to induce the characteristics of ER stress; mitochondrial Ca^2+^ overloading, enhanced ER staining, release of glucose-regulated protein 78 (GRP78), phosphorylation of RNA-dependent protein kinase (PKR) like ER kinase (PERK) and eukaryotic initiation factor-2*α* (eIF-2*α*), cleavage of activating transcription factor 6 (ATF6) and caspase 12, and upregulation of CCAAT/enhancer-binding protein-homologous protein (CHOP). However, KIOM-4 attenuated these changes induced by STZ. Furthermore, KIOM-4 suppressed apoptosis induced by STZ in CHOP downregulated cells using CHOP siRNA. These results suggest that KIOM-4 exhibits protective effects in STZ-induced pancreatic *β*-cell damage, by interrupting the ER stress-mediated pathway.

## 1. Introduction

KIOM-4 is a combination of extracts obtained from *Magnolia officinalis, Pueraria lobata, Glycyrrhiza uralensis,* and *Euphorbia pekinensis. Magnolia officinalis* exhibits anticancer, antimutagenic, hepatoprotective, neuroprotective, antiinflammatory and antimicrobial activities [1–7]. *Pueraria lobata *possesses antiatherogenic, antimutagenic, antidiabetic, and antioxidant properties [8–11]. *Glycyrrhiza uralensis *possesses detoxifying, antioxidant [12], antiulcer, antiinflammatory, antiviral, antiatherogenic, anticarcinogenic properties [13], and cytoprotective effect against hepatotoxicity [14]. *Euphorbia pekinensis *is widely distributed in northeast mainland China and its roots have been used as traditional folk medicine for the treatment of edema, and shown to possess antiviral and cytotoxic properties [15, 16]. We recently demonstrated that KIOM-4 exhibits cytoprotective effects against streptozotocin- (STZ-) induced oxidative stress damage in *β*-cells through catalase activation [17] and heme oxygenase-1 [18], and protects cells against STZ-induced mitochondrial damage in pancreatic *β*-cells through its antioxidant properties [19].

Type 1 diabetes results from autoimmune and/or inflammatry processes that selectively disrupt insulin-producing pancreatic *β*-cell, and is characterized by hyperglycemia due to reduced insulin secretion. Apoptosis is considered to be the main mode of pancreatic *β*-cell death in diabetes [20, 21]. One of the characteristic features of *β*-cells is a highly developed endoplasmic reticulum (ER), apparently due to heavy engagement in insulin secretion, as the early steps of insulin biosynthesis occur in the ER [22, 23]. ER is the site for folding and assembling proteins, lipid biosynthesis, vesicular traffic, and cellular calcium storage, and is sensitive to alterations in homeostasis. Proper ER function is essential to cell survival and perturbation of its function induces cellular damage and results in apoptosis. Various conditions can disturb ER functions and these conditions include inhibition of protein glycosylation, reduction of disulfide bond formation, calcium depletion from the ER lumen, impairment of protein transport from the ER to the Golgi apparatus, and expression of misfolded proteins. Such dysfunction causes proteotoxicity in the ER, collectively termed ER stress [24–26]. Recent studies suggest that ER stress plays an important role in the loss of *β*-cells [26–28]. Given that ER is a unique oxidizing folding-environment that favors disulfide bond formation, any change in cell redox state can triggers ER stress and parallel antioxidants protect cells from ER stress [29]. Furthermore, it has been suggested that the accumulation of misfolded protein within the lumen of the ER leads to ER stress, which in turn causes oxidative stress and finally cell death [30]. Recent research also revealed that antioxidants, such as butylated hydroxyanisole, could reduce ER stress [31]. Therefore, in present study, we hypothesized that the cytoprotective mechanism of KIOM-4 against STZ-induced pancreatic *β*-cells damage may be involved in interrupting ER stress-mediated apoptotic pathway.

## 2. Materials and Methods

### 2.1. Preparation of KIOM-4

The KIOM-4 was provided by Jin Sook Kim PhD of the Korea Institute of Oriental Medicine (Daejeon, Korea). It was dissolved in dimethyl sulfoxide (DMSO), the final concentration of which did not exceed 0.1%.

### 2.2. Reagents

STZ was purchased from Calbiochem (San Diego, CA). Hoechst 33342 and [3-(4,5-dimethylthiazol-2-yl)-2,5-diphenyltetrazolium] bromide (MTT) were purchased from Sigma Chemical Company (St. Louis, MO). ER-Tracker Blue-White DPX and Rhod-2 acetoxymethyl ester (Rhod-2 AM) were purchased from Molecular Probes (Eugene, OR). Anti-CCAAT/enhancer-binding protein-homologous protein (CHOP), -capsase 12, -activating transcription factor 6 (ATF6), and *β*-actin antibodies were purchased from Cell Signaling Technology (Beverly, MA). Antiphospho eukaryotic initiation factor-2*α* (eIF-2*α*), glucose-regulated protein 78 (GRP78), and phospho-RNA-dependent protein kinase (PKR) like ER kinase (PERK) antibodies were purchased from Santa Cruz Biotechnology (Santa Cruz, CA). The other chemicals and reagents were of analytical grade.

### 2.3. Cell Culture

RINm5F rat pancreatic *β*-cells were maintained at 37°C in an incubator with a humidified atmosphere of 5% CO_2_, and cultured in RPMI 1640 medium containing 10% heat-inactivated fetal calf serum, streptomycin (100 *μ*g/mL), and penicillin (100 units/mL).

### 2.4. Cell Viability Assays

Cells were seeded in a 96 well plate at a concentration of 1 × 10^5^ cells/mL and were treated with KIOM-4 at 50 *μ*g/mL. After 1 hour, 10 mM of STZ was added, and the mixture was incubated for 24 hours. Fifty *μ*L of the MTT stock solution (2 mg/mL) was then added into each well to attain a total reaction volume of 200 *μ*L. After incubating for 4 hours, the plate was centrifuged at 800 × *g* for 5 minutes, and the supernatants were aspirated. The formazan crystals in each well were dissolved in 150 *μ*L of DMSO and read at A_540_ on a scanning multiwell spectrophotometer [32, 33].

### 2.5. Nuclear Staining with Hoechst 33342

Cells were placed in a 24 well plate at 2 × 10^5^ cells/well. At 16 hours after plating, cells were treated with 50 *μ*g/mL of KIOM-4 and after further incubation for 1 hour, 10 mM STZ was added to the culture. After 24 hours, 1.5 *μ*L of Hoechst 33342 (stock 10 mg/mL), a DNA-specific fluorescent dye, was added to each well (final 15 *μ*g/mL) and incubated for 10 minutes at 37°C. The stained cells were then observed under a fluorescent microscope, which was equipped with a CoolSNAP-Pro color digital camera, to examine the extent of nuclear condensation.

### 2.6. DNA Fragmentation

Cellular DNA fragmentation was assessed by cytoplasmic histone-associated DNA fragmentation kit from Roche Diagnostics (Mannheim, Germany) according to the manufacturer's instructions.

### 2.7. Mitochondrial Ca^2+^ Measurements

Rhod-2 AM probe was used to determine mitochondrial Ca^2+^ level [34]. Rhod-2 AM has a net positive charge, which facilitates its sequestration into mitochondria due to membrane potential-driven uptake. The use of Rhod-2 AM enhances the selectivity for mitochondrial loading, because this dye exhibits Ca^2+^-dependent fluorescence only after it is oxidized, and this occurs preferentially within the mitochondria. Cells were seeded in a 6 well plate at 1 × 10^5^ cells/mL and were treated with KIOM-4 at 50 *μ*g/mL. After 1 hour, 10 mM of STZ was added, and the mixture was incubated for 24 hours. Cells were harvested, washed, and suspended in PBS containing Rhod-2 AM (1 *μ*M). After 15 minutes of incubation at 37°C, the cells were washed, suspended in PBS, and analyzed by flow cytometry.

### 2.8. Fluorescent Microscopy and Staining

Image analysis for ER staining was achieved by seeding cells on a cover-slip loaded six well plate at 1 × 10^5^ cells/mL. Sixteen hours after plating, cells were treated with KIOM-4. One hour following KIOM-4 treatment, STZ at 10 mM, thapsigargin at 2 *μ*g/mL, or tunicamycin at 2 *μ*g/mL was added to the plates, respectively. Twenty four hours later, ER-Tracker Blue-White DPX probe was added to the cells and incubated for 30 minutes under the same growth conditions. The loading solution was removed and cells were then washed with PBS. Microscopic images were collected using the Laser Scanning Microscope 5 PASCAL program (Carl Zeiss, Jena, Germany) on a confocal microscope.

### 2.9. Western Blot Analysis

Aliquots of the lysates (40 *μ*g of protein) were boiled for 5 minutes and electrophoresed on a 10% SDS-polyacrylamide gel. Blots in the gels were transferred onto nitrocellulose membranes (Bio-Rad, Hercules, CA), which were then incubated with primary antibodies. The membranes were further incubated with secondary antibody-horseradish peroxidase conjugates (Pierce, Rockland, IL), and exposed to X-ray film. Protein bands were detected using an enhanced chemiluminescence Western blotting detection kit (Amersham, Little Chalfont, Buckinghamshire, UK).

### 2.10. Transient Transfection of Small RNA Interference (siRNA)

Cells were seeded at 1.5 × 10^5^ cells/well in 24 well plate and allowed to reach approximately 50% confluence on the day of transfection. The siRNA construct used was obtained as mismatched siRNA control (siControl, Santa Cruz Biotechnology, Santa Cruz, CA) and siRNA against CHOP (siCHOP, Bioneer Corporation, Bioneer, South Korea). Cells were transfected with 10–50 nM siRNA using lipofectamineTM 2000 (Invitrogen, Carlsbad, CA) according to the manufacturer's instruction. At 24 hours after transfection, cells were treated with or without KIOM-4 and/or STZ for 24 hours and examined by Hoechst 33342 staining and DNA fragmentation, respectively.

### 2.11. Statistical Analysis

All the measurements were made in triplicate and all values are represented as the mean ± standard error of the mean (SEM). Data were subjected to an analysis of the variance (ANOVA) using the Tukey test to analyze the difference. *P* < .05 was considered statistically significant.

## 3. Results

### 3.1. Attenuated Characteristics of Apoptosis in RINm5F Cells Exposed to STZ by KIOM-4 Treatment

**Figure 1:**
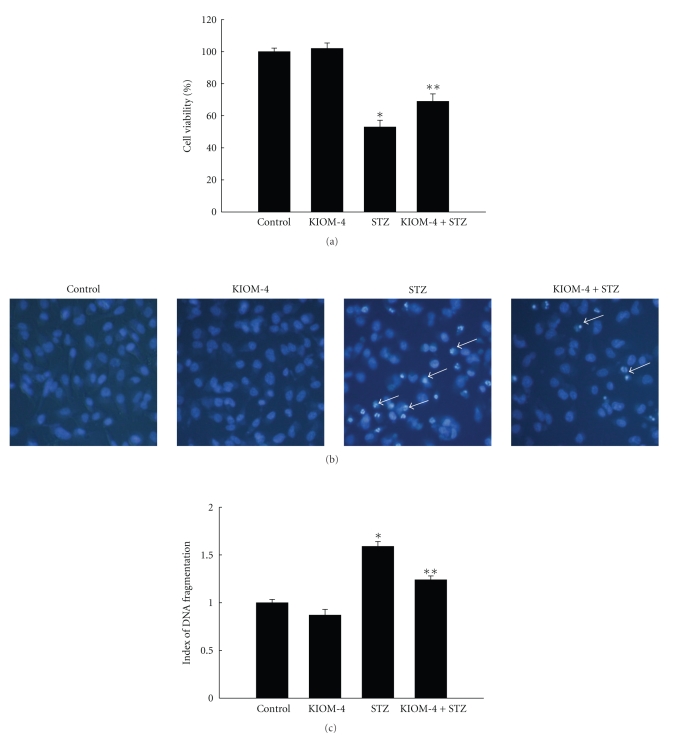
The effects of KIOM-4 on STZ-induced cell damage. RINm5F cells were treated with KIOM-4 at 50 *μ*g/mL, after 1 hour, 10 mM of STZ was added and was incubated for 24 hours. (a) Cell viability was measured using MTT assay. *Significantly different from control cells (*P* < .05). **Significantly different from STZ-treated cells (*P* < .05). (b) Apoptotic body formation was observed under a fluorescence microscope after Hoechst 33342 staining (original magnification, x400). Arrows indicate apoptotic bodies. (c) DNA fragmentation was quantified with an ELISA kit. Measurements were made in triplicate and the values are expressed as means ± SEM. *Significantly different from control cells (*P* < .05) and **significantly different from STZ-treated cells (*P* < .05).

To investigate the potential role of KIOM-4 on STZ induced *β*-cells damage, we first examined the effects of KIOM-4 on cell survival. As shown in Figure 1(a), KIOM-4 significantly inhibited STZ-induced cell damage. In addition, apoptosis was determined by morphological microscopy after staining of cellular nuclei with Hoechst 33342. The microscopic images in Figure 1(b) indicate that the control cells retained intact nuclei, while STZ-treated cells showed significant nuclear fragmentation, which is characteristic of apoptosis, while KIOM-4 reduced it. STZ dramatically increased the levels of DNA fragmentation compared to control, while KIOM-4 treatment attenuated STZ-induced DNA fragmentation (Figure 1(c)). Taken together, these results indicate that KIOM-4 protects cell viability by inhibiting STZ- induced apoptosis.

### 3.2. Reduction of STZ-Induced Mitochondrial Ca^2+^ Overloading and ER Stress by KIOM-4 Treatment

**Figure 2:**
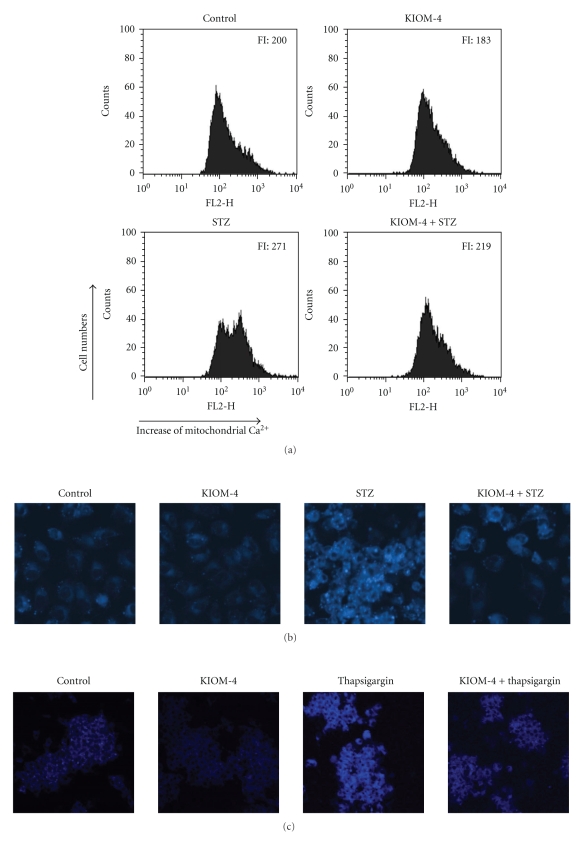
The effects of KIOM-4 on STZ-induced mitochondrial Ca^2+^ overloading and ER staining. Cells were treated with fluorescent probe Rhod-2 AM. The mitochondrial Ca^2+^ levels were measured by (a) flow cytometry as described in Section 2. FI indicates the fluorescence intensity of Rhod-2 AM. ((b) and (c)) Cells were treated with ER Tracker Blue-White DPX for ER staining as describe in Section 2.

Depletion of ER calcium stores can induce ER stress [35], which leads to an increase in mitochondrial Ca^2+^ [36]. We assessed the effects of KIOM-4 on mitochondrial Ca^2+^ mobilization. As shown in Figure 2(a), STZ treatment resulted in a significant increase in mitochondrial Ca^2+^ levels at 24 hours, while KIOM-4 treatment reduced STZ-induced mitochondrial Ca^2+^ overloading. Thapsigargin, which selectively inhibits the Ca^2+^-ATPase responsible for Ca^2+^ accumulation of ER, and tunicamycin, which blocks N-linked protein glycosylationare, are well known for their capacity to induce ER stress [37, 38]. Recently, ER stress by thapsigargin has been shown to increase fluorescent intensity of the dye following ER staining with ER-Tracker Blue-White DPX [39]. KIOM-4 significantly attenuated the staining intensity of this dye increased by STZ treatment (Figure 2(b)). In our study, KIOM-4 also reduced the fluorescent intensity of ER staining, which is induced by thapsigargin (Figure 2(c)), while KIOM-4 did not show the capacity to reduce the fluorescent intensity increased by tunicamycin (data not shown).

**Figure 3:**
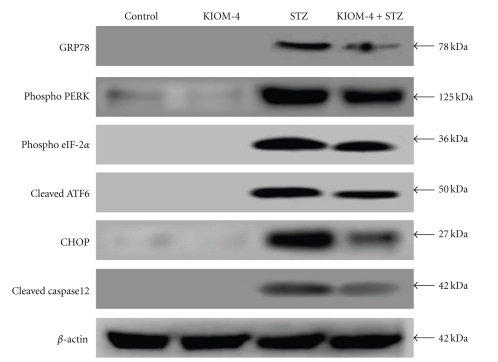
The effects of KIOM-4 on STZ-induced ER stress. The expression of GRP78, PERK and its downstream eIF-2*α* phosphorylation as well as cleaved ATF6, capsase 12, and CHOP were determined by western blotting analysis.

Increased PERK phosphorylation and upregulation of GRP78 are major indicators of ER stress [35]. eIF-2*α* is phosphorylated by PERK in response to ER stress, leading to attenuation of translational initiation and protein synthesis [40, 41]. Therefore, we examined whether the effects of KIOM-4 on STZ-induced *β*-cell damage is associated with the GRP78 and PERK/eIF-2*α* kinase signaling pathway. STZ treatment induced a dramatic increase in GRP78, phosphorylated PERK and eIF-2*α* levels, and KIOM-4 attenuated these changes (Figure 3). Another hallmark of ER stress responses include ATF6 activation (cleaved form of ATF6) and subsequent induction of CHOP [42, 43]. STZ enhanced ATF6 activation and induced CHOP expression, while KIOM-4 abolished these changes (Figure 3). Caspase 12 has been reported to become activated during ER stress [35]. KIOM-4 treatment also attenuated STZ induced activation of caspase 12 (cleaved form of caspase 12) following ER stress (Figure 3). These results suggest that ER stress-mediated apoptosis was involved in the STZ-treated cells, and KIOM-4 inhibited these changes.

### 3.3. Suppression of Cell Death Induced by STZ in CHOP Downregulated Cells by KIOM-4 Treatment

**Figure 4:**
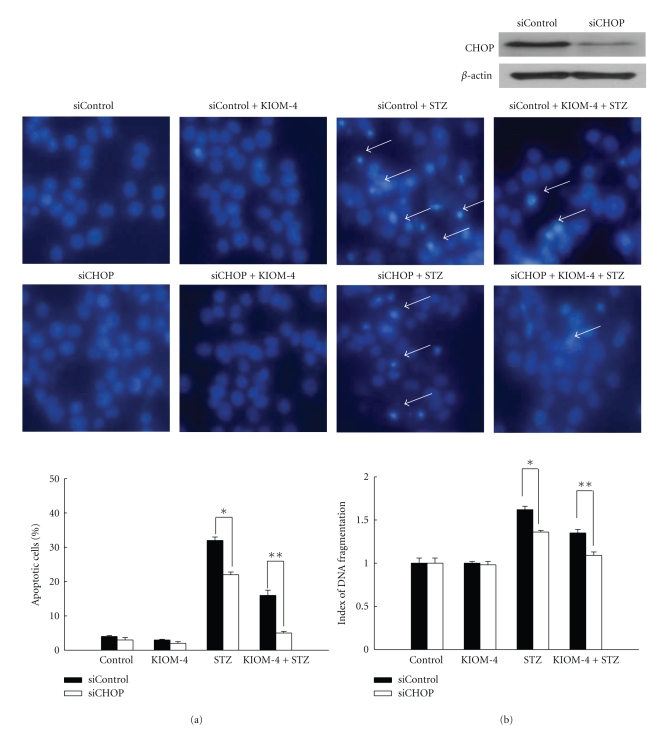
The effects of KIOM-4 on cell death induced by STZ in CHOP-downregulated cells. (a) Cells were transfected with CHOP siRNA or control siRNA, the transfected cells were treated with KIOM-4 at 50 *μ*g/mL. After 1 hour, 10 mM of STZ was added and further incubated for 24 hours. (a) Apoptotic body formation was observed under a fluorescence microscope and quantitated after Hoechst 33342 staining (original magnification x400). Arrows indicate apoptotic bodies. *Significantly different between STZ-treated siControl and STZ-treated siCHOP (*P* < .05) and **significantly different between KIOM-4 plus STZ-treated siControl and KIOM-4 plus STZ-treated siCHOP cells (*P* < .05). (b) DNA fragmentation was quantified with an ELISA kit. Measurements were made in triplicate and values are expressed as means ± SEM. *Significantly different between STZ-treated siControl and STZ-treated siCHOP (*P* < .05), and **significantly different between KIOM-4 plus STZ-treated siControl and KIOM-4 plus STZ-treated siCHOP cells (*P* < .05).

CHOP is reported to play a proapoptotic role during ER stress [42]. To investigate whether CHOP is directly involved in STZ-induced apoptosis, we employed siRNA against CHOP mRNA. As shown in Figures 4(a) and 4(b), suppressed CHOP expression were shown to further alleviate apoptotic phenomena in STZ-treated or KIOM-4 plus STZ-treated siCHOP cells, compared to STZ-treated or KIOM-4 plus STZ-treated siControl cells. These results suggest that CHOP may be in part involved in STZ-induced apoptosis which is mediated by ER stress pathway.

## 4. Discussion

ER stress plays a key role in the pathogenesis of diabetes, contributing to pancreatic *β*-cell loss and insulin resistance [20]. Baseline of ER stress in *β*-cells is higher than in other cell types, because of their exposure to frequent energy fluctuations and the early steps of insulin biosynthesis occur in ER [22, 23]. Numerous studies have reported that antioxidant treatment, which targets oxidative stress, may help prevent or delay the development of diabetes and its complications [44, 45]. In our study, KIOM-4 exhibited antioxidant effects by activating catalase, heme oxygenase-1, and manganese superoxide dismutase [17–19]. In this context, the antidiabetic effects of KIOM-4 on ER stress induced by STZ treatment in pancreatic *β*-cells were elucidated. Because elevation of mitochondrial Ca^2+^ levels or depletion of ER Ca^2+^ stores is involved in ER stress-mediated apoptosis, we revealed that STZ-induced mitochondrial Ca^2+^ overloading, indicating that alteration in Ca^2+^ homeostasis is implicated in STZ-induced apoptosis, while KIOM-4 treatment restored STZ-induced mitochondrial Ca^2+^ overloading. Moreover, recent studies suggest that increasing the fluorescent intensity following ER staining with ER-Tracker Blue-White DPX was considered a character of ER stress [37, 46]. In our study, STZ significantly increased the staining intensity of this dye, while KIOM-4 attenuated the increased staining intensity. We further investigated specific markers of ER stress. The unfolded protein response of mammalian cells is initiated by three ER transmembrane proteins; PERK, IRE1, and ATF6. Many ER resident proteins display altered expression pattern under ER stress. Under unstressed conditions, the luminal domains of these sensors are occupied by the ER chaperon GRP78 [47–49]. Upon ER stress, sequestration of GRP78 by unfolded proteins activates these sensors by inducing phosphorylation and homodimerization of PERK and IRE1, and relocation and proteolytic cleavage of ATF6 [45–47]. These three ER stress sensors trigger divergent and convergent signaling cascades, leading to adaptation or cell death [50]. Our study found that STZ-induced GRP78 release, PERK phosphorylation, and ATF6 cleavage while KIOM-4 inhibited these altered expression pattern in ER stress. Therefore, we speculated that the suppression of PERK phosphorylation and ATF6 cleavage by KIOM-4 treatment was mainly due to inhibition of GRP78 release. eIF-2*α* is phosphorylated by PERK in response to ER stress, leading to attenuation of translational initiation and protein synthesis [40, 41]. We also revealed that KIOM-4 attenuated STZ-induced eIF-2*α* phosphorylation. Caspase-12 has been reported to become activated during ER stress [35]. STZ treatment increases the activation of caspase-12 located in the cytoplasmic side of ER, while KIOM-4 inhibited caspase-12 activation [35]. CHOP protein was first identified to be a member of the CCAAT/enhancer binding proteins (C/EBPs) that dimerize with transcription factors C/EBP and Liver-enriched activation protein, and functions as a dominant-negative inhibitor of gene transcription [42]. CHOP is either not expressed or is expressed at low levels under physiological conditions, but is strongly induced in response to ER stress at the level of transcription [51]. Over-expression of CHOP leads to growth arrest and apoptosis [52–54]. CHOP knockout mice show normal development and fertility, but exhibit reduced apoptosis in response to ER stress [55–57]. Therefore, CHOP plays an important role in the induction of ER stress-mediated apoptosis [55, 58]. We observed that suppression of CHOP by CHOP siRNA attenuated STZ-induced apoptosis, suggesting that ER stress may be of importance for the cytotoxic activity of STZ. KIOM-4 treatment further reversed STZ-induced apoptosis in CHOP siRNA transfected cells, compared to control siRNA transfected cells. Hence, these results suggest that KIOM-4 protects against STZ-induced apoptosis in diabetic pancreatic *β*-cells via inhibition of ER stress.

**Figure 5:**
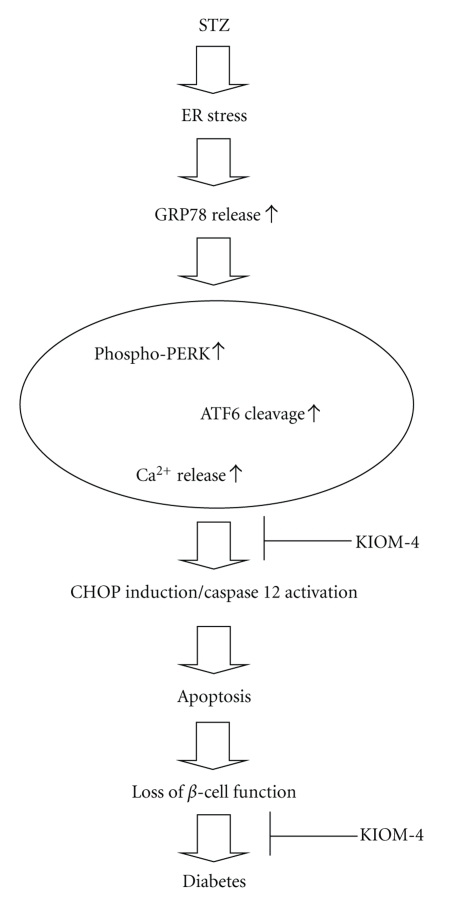
Model illustrating the inhibitory effect of KIOM-4 against STZ-induced ER stress pathway.

Taken together, this study demonstrated that KIOM-4 protected against STZ-induced *β*-cell damage via inhibition of ER stress-mediated apoptosis (Figure 5).
